# Serum-based BRAF V600E detection improves early diagnosis of papillary thyroid carcinoma: A prospective diagnostic accuracy study compared with ultrasound-guided cytology: Erratum

**DOI:** 10.1097/MD.0000000000050152

**Published:** 2026-08-07

**Authors:** Qi Li, Yiyan Lu, Xiangli Li, Jing Jiang, Xiuqing Wang, Jia Xu, Qi Zhang, Longzhu Zhao

In the article “Serum-based BRAF V600E detection improves early diagnosis of papillary thyroid carcinoma: A prospective diagnostic accuracy study compared with ultrasound-guided cytology,”^[1]^ which appears in Volume 105, Issue 24 of *Medicine*, the ethical approval number has been changed in the online version from “BITC2025” to “ZXYEC-LW-2026-10.” This change affects the sentence “The study protocol was approved by the Ethics Committee of Beijing Integrated Traditional Chinese and Western Medicine Hospital (Approval number: BITC2025)” both in the 1st page footnote and in subsection 2.1, 1st paragraph.
